# APEX1 regulates alternative splicing of key tumorigenesis genes in non-small-cell lung cancer

**DOI:** 10.1186/s12920-022-01290-0

**Published:** 2022-07-02

**Authors:** Li Peng, Yuwei Liu, Jing Chen, Mengxin Cheng, Ying Wu, Min Chen, Ya Zhong, Dan Shen, Ling Chen, Xujun Ye

**Affiliations:** 1 Department of Internal Medicine and Geriatrics, Zhongnan Hospital of Wuhan University, Wuhan University, No. 169 Dong Hu Road, Wuhan, 430071 Hubei China; 2 Department of Cardiology, Zhongnan Hosipital of Wuhan University, Wuhan University, Wuhan, 430071 Hubei China

**Keywords:** APEX1, RNA-seq, Alternative Splicing, Non-Small-Cell Lung Cancer (NSCLC)

## Abstract

**Background:**

Aberrant alternative splicing (AS) contributes to tumor progression. Previous studies have shown that apurinic-apyrimidinic endonuclease-1 (APEX1) is involved in tumor progression. It is unknown whether APEX1 functions in tumor progression by regulation of AS. It is also unknown whether APEX1 can regulate non-small-cell lung cancer (NSCLC) proliferation and apoptosis. We analyzed APEX1 expression levels in 517 lung NSCLC samples from the TCGA (Cancer Genome Atlas) database. The impact of APEX1 over expression on A549 cell proliferation and apoptosis was detected by the methyl thiazolyl tetrazolium assay and by flow cytometry. The transcriptome of A549 cells with and without APEX1 over expression was determined by Illumina sequencing, followed by analysis of AS. RT-qPCR validated expression of APEX1-related genes in A549 cells. We have successfully applied RNA-seq technology to demonstrate APEX1 regulation of AS.

**Results:**

APEX1 expression was shown to be upregulated in NSCLC samples and to reduce cell proliferation and induce apoptosis of A549 cells. In addition, APEX1 regulated AS of key tumorigenesis genes involved in cancer proliferation and apoptosis within MAPK and Wnt signaling pathways. Each of these pathways are involved in lung cancer progression. Furthermore, validated AS events regulated by APEX1 were in key tumorigenesis genes; AXIN1 (axis inhibition protein 1), GCNT2 (N-acetyl glucosaminyl transferase 2), and SMAD3 (SMAD Family Member 3). These genes encode signaling pathway transcription regulatory factors.

**Conclusions:**

We found that increased expression of APEX1 was an independent prognostic factor related to NSCLC progression. Therefore, APEX1 regulation of AS may serve as a molecular marker or therapeutic target for NSCLC treatment.

**Supplementary Information:**

The online version contains supplementary material available at 10.1186/s12920-022-01290-0.

## Introduction

With increasing worldwide incidence and mortality, lung cancer has become the major cause of cancer death and a serious clinical issue [1]. Non-small-cell lung cancer (NSCLC) is the most prevalent and heterogeneous subtype of lung cancer comprised of adenocarcinoma, squamous cell carcinoma, and large cell carcinoma [2]. NSCLC is often is often diagnosed at an advanced stage, leaving patients with little chance for effective and curative treatment, with a 5-year survival rate of only 0.15% [3]. Fortunately, important advances have been achieved in the treatment of NSCLC over the past two decades [4]. The clinical treatments available in routine practice for NSCLC patients include molecularly targeted therapeutics, immune checkpoint inhibitors, and anti-angiogenic agents, which have significantly improved patient outcomes [5]. Therefore, improved patient prognosis and survival requires improvements in screening for early NSCLC biomarkers, development of therapeutic efficacy predictors, and new treatment drugs.

Data from genome-wide studies suggest that more than 90% of human genes undergo alternative splicing (AS) [6]. Evidence suggests that AS contributes to cell differentiation, lineage determination, tissue-identity acquisition, tissue maintenance, and organ development [7]. While, systematic and coordinated AS alterations of functionally linked RNA-binding proteins (RBPs) could impact cell carcinogenesis [8]. RBPs are important regulators of AS and have important roles: in cancer progression, they are key components of RNA metabolism of RNAs. Using mass spectrometry, more than 1000 RBPs have been identified in eukaryotic cells, many of which regulate splicing [9–11]. Thus, exploration of RBP regulatory mechanisms associated with AS will deepen our understanding of cancer. Altered expression of apurinic-apyrimidinic endonuclease-1 (APEX1), known as an RBP, is often observed in human tumors [12]. Accumulating evidence indicates that elevated and ectopic expression of APEX1 in tumor tissue is closely linked to a poor prognosis for lung cancer [12, 13].

Although previous studies have suggested involvement of APEX1 in tumor progression, it remains unclear whether APEX1 regulates AS in NSCLC. The aim of this study was to determine whether APEX1 indirectly regulated proliferation and apoptosis of NSCLC through AS. To accomplish this aim, we assessed the relationships among APEX1, cellular proliferation, apoptosis regulation, and cancer progression through specific signaling pathways. The approach was to analyze APEX1 expression levels in 517 lung NSCLC samples from TCGA (The Cancer Genome Atlas) database and as well, the impact of APEX1 on A549 cell proliferation and apoptosis. Transcriptomes of A549 cells with and without APEX1 overexpression were evaluated by Illumina sequencing, followed by analysis of AS regulation. Reverse transcription-quantitative polymerase chain reaction (RT-qPCR) was performed to validate APEX1 in A549 cells and clinical NSCLC samples. We found that APEX1 expression was upregulated in NSCLC samples, and that APEX1 overexpression led to reduced cell proliferation and apoptosis. Furthermore, APEX1-regulated AS of many genes, which were related to cancer proliferation and apoptosis pathways.

## Materials and methods

### Cloning and plasmid construction

The cloning and plasmid construction performed as previously reported [9]. The human APEX1 gene was cloned by reverse transcription from total RNAs extracted from multiple cancer and non-cancer cell lines, followed by PCR amplification. The DNA fragment corresponding to a complete cDNA length was purified from a gel (MinElute PCR Purification Kit, Qiagen, 28004), and then cloned into the pIRES-hrGFP-1a vector (240031, Agilent Technologies) using the hot fusion method were designed with CE Design V1.04 (Vazyme Biotech Co., Ltd). Each of the primer comprises a fragment of gene specific sequence and a 17–30 bp sequence of the pIRES-hrGFP-1a vector. Please be noted that this vector also harbored a FLAG tag, which was fused to the 3’ end of APEX1. The primers used for cDNA cloning were as follow,

- APEX1 F Primer: agcccgggcggatccgaattcATGCCGAAGCGTGGGAAA
- APEX1 R Primer: gtcatccttgtagtcctcgagCAGTGCTAGGTATAGGGTGATAGG

The pIRES-hrGFP-1a vector was digested by EcoRI (NEB, 3101S) and XhoI (NEB R0146V) at 37 ℃ for 2–3 h. Then enzyme-digested vector was run on 1.0% agarose gel and purified by Qiagen column kit (MinElute PCR Purification Kit, Qiagen, 28004). Then the insert fragment was synthesized by PCR amplification and PCR insert were added to a PCR microtube for ligation (T4 DNA ligase, NEB, M0202V) with ClonExpress® II One Step Cloning Kit (Vazyme, C112). Plasmids were introduced into Escherichia coli strain by chemical transformation. Cells were plated onto LB agar plates containing 1 µL/ml ampicillin (sigma, 7177-48-2), and incubated overnight at 37 ℃. Colonies were screened by colony PCR (28 cycles) with universal primers (located on the backbone vector). The insert sequence was verified by Sanger sequencing.

### RNA extraction and sequencing

Before extracting RNA, A549 cells were ground to a fine powder. Total RNA was extracted with TRIzol (Life Technology, Carlsbad, CA, USA). The RNA was further purified with two phenol–chloroform treatments. The quality and quantity of purified RNA was measured at 260 nm/280 nm (A260/A280) using a Smartspec Plus (BioRad, Sacramento, CA, USA). The integrity of RNA was further verified by 1.5% agarose gel electrophoresis. For each sample, 1 µg of total RNA was used for RNA-seq library preparation with a VAHTS Stranded mRNA-seq Library Prep Kit (Vazyme, Nanjing, China). For high-throughput sequencing, the library was prepared according to the manufacturer's instructions and analyzed with the Illumina HiSeq X Ten system for 150 nt paired-end sequencing.

### Assessment of APEX1 overexpression

GAPDH (glyceraldehyde-3-phosphate dehydrogenase) was used as a control gene to assess the effects of APEX1 overexpression. The cDNA synthesis was completed by standard procedures and RT-qPCR was performed with a Bio-Rad S1000 with Bestar SYBR Green RT-PCR master mix (DBI Bioscience, Shanghai, China). Primer information is provided in Additional file 1: Table S1.

### Western blotting analysis

The western blotting analysis performed as previously reported [9]. A549 cells line after 48 h transfection were collected in each group, lysed on ice for 30 min in RIPA buffer containing 50 mM Tris–HCl (pH 7.4), 150 mM NaCl, 1.0% deoxycholate, 1% Triton X-100, 1 mM EDTA and 0.1% SDS. The samples were centrifuged (12,000 rpm, 5 min), and supernatants were stored separately. The protein was stored and frozen in refrigerator at 4 °C after the protein concentration of every sample. Protein samples were loaded into 10% or 12% SDS-PAGE gels depending on molecular weight and transferred onto 0.45 mm PVDF membranes. Then incubated with horseradish peroxidase-conjugated secondary antibody for 1 h at room temperature, and membranes were visualized through chemiluminescence. We also have quantitated some of the WB bands by the software Image J. Antibodies: The following antibodies were purchased from commercial sources including anti-APEX1 (Polyclonal Antibody, AB clonal; ab268072); anti-GAPDH (Polyclonal Antibody, AB clonal; AC001).

### RNA-Seq raw data cleaning and alignment

Original reads containing more than 2-N bases were discarded. Then adaptors and low-quality bases were trimmed from raw sequencing reads using the FASTX-Toolkit (Version 0.0.13). Short reads less than 16 nt were deleted. After that, tophat2 [14] aligned the clean reads with the GRch38 genome, allowing four mismatches. Uniquely mapped reads were used for gene read counts and FPKM calculations (fragments per kb of transcripts per million fragments mapped).

### Differentially expressed genes (DEGs) analysis and alternative splicing analysis

The R Bioconductor package edgeR was utilized to identify the differentially expressed genes (DEGs). Alternative splicing events (ASEs) and regulated alternative splicing events (RASEs) between the samples were defined and quantified using the ABL pipeline [15]. In short, the detection of ABL, as 10 types of ASEs, was based on splice junction reads, including exon skipping (ES), alternative 5' splice site (A5SS), alternative 3'splice site (A3SS), intron retention (IR), mutually exclusive exons (MXE), mutually exclusive 5'UTRs (5pMXE), mutually exclusive 3'UTRs (3pMXE), cassette exon, A3SS&ES, and A5SS&ES. Student’s *t*-test was performed to evaluate the ASE regulated by RNA-binding proteins. The events that were significant at the cut-off *p* value (corresponding to the cut-off of the 5% false discovery rate) were considered ASE for RBP control.

### Reverse transcription qPCR and validation of DEGs and AS events

To validate the RNA-seq data, qRT-PCR was performed on some DEGs and ASE. Primer information is found in Additional file 1: Table S1. According to the manufacturer's instructions, real-time PCR was performed by SYBR Green PCR Reagents Kit (Yeasen, Shanghai, China). To detect alternative isoforms, we used crossover primers. Design boundary-spanning primers for alternative exons according to “model exons” was used to detect model splicing, or “altered exons” to detect altered splicing.

### Functional enrichment analysis and downloading RNA-seq data for NSCLC samples

To assess the functional categories of DEGs, Gene Ontology (GO) terms and Kyoto Encyclopedia of Genes and Genomes (KEGG) pathways were identified with a KOBAS 2.0 server [16–18]. The Hypergeometric test and the Benjamini–Hochberg FDR controlling procedure were used to define enrichment terms. RNA-seq data for NSCLC samples were downloaded from the Cancer Genome Atlas (TCGA) database for analysis of APEX1 expression and regulation of AS in NSCLC. The data presented in this publication are available using GEO Series accession number GSE146875.

### Statistical analysis

All experiments were repeated three times and presented as the mean ± standard deviation. The analysis was performed by GraphPad Prism 7 software (GraphPad, Inc. San Diego, CA, USA) and SPSS version 21.0 software (SPSS, Inc., Chicago, IL, USA). Student's *t*-test was used to analyze two groups. One-way analysis of variance followed by the Newman-Keuls test was used to compare the differences among multiple groups. All *p* values were two-sided, with the statistically significant level defined as lower than 0.05.

## Results

### APEX1 expression is upregulated during all NSCLC stages

**Fig. 1:**
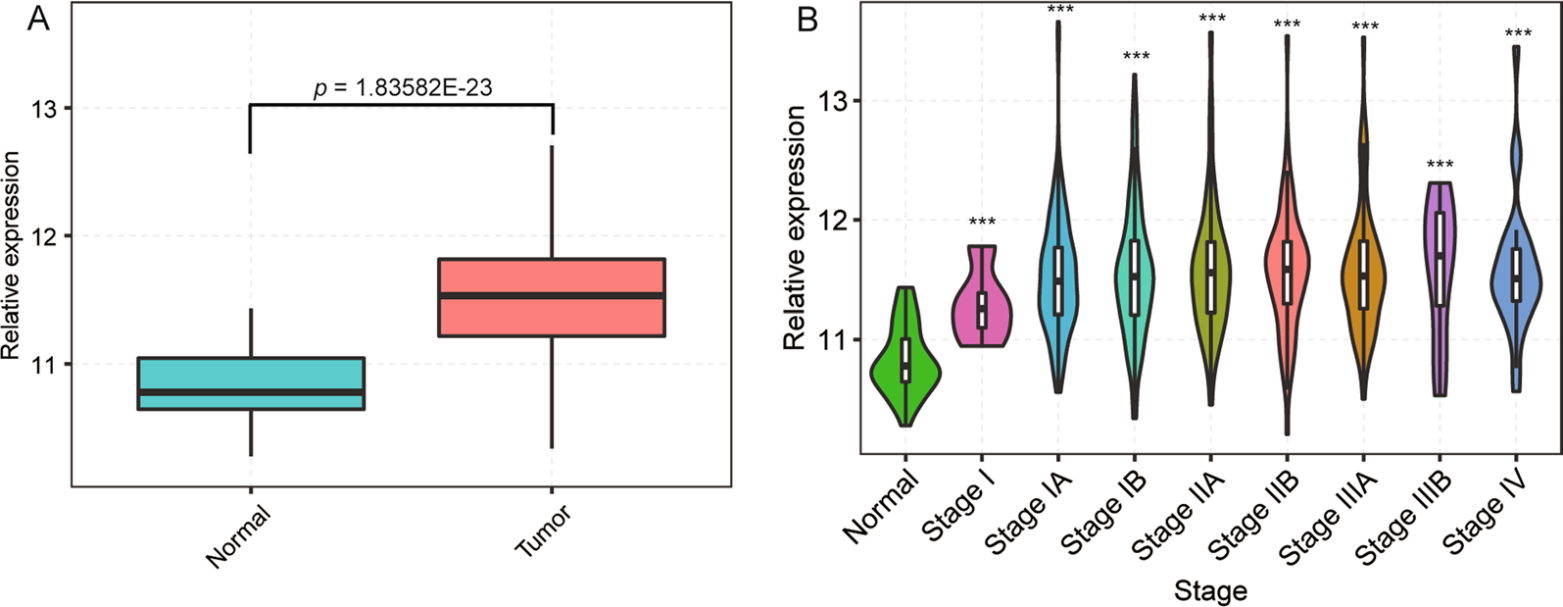
APEX1 expression in NSCLC samples based on the TCGA database. **a** Column chart of APEX1 expression in 517 NSCLC samples and 59 normal samples. **b** APEX1 distribution of expression levels during different stages of NSCLC samples. NSCLC was staged based on the standards of the International Association for Lung Cancer Research (IASLC) stage (log-rank test, ***p < 0.001, compared with normal samples)

In order to investigate APEX1 differential expression and clinical relevance for all NSCLC stages, we used the clinical and transcriptomic data from the TCGA database (https://cancergenome.nih.gov/), which included 517 NSCLC and 59 normal samples. We found that APEX1 was upregulated in 517 NSCLC samples compared to 59 normal tissue samples, with a statistically significant difference between the two groups (*p* < 0.01, Fig. 1a and Additional file 2: Table S2). In addition, 517 NSCLC samples were classified into different stages, including stage I (5), IA (132), IB (140), IIA (50), IIB (71), IIIA (73), IIIB (11) and IV (26) (Fig. 1b). It was showed that APEX1 was significantly upregulated in most stages compared with the normal stage (*p* < 0.01). Collectively, these data demonstrated APEX1 to be upregulated and its levels to be correlated with malignant features of human NSCLCs. Therefore, it was necessary to study the intrinsic mechanism of APEX1 on cancer cell proliferation or apoptosis.

### Overexpression of APEX1 induces apoptosis and inhibits proliferation of A549 cells

**Fig. 2:**
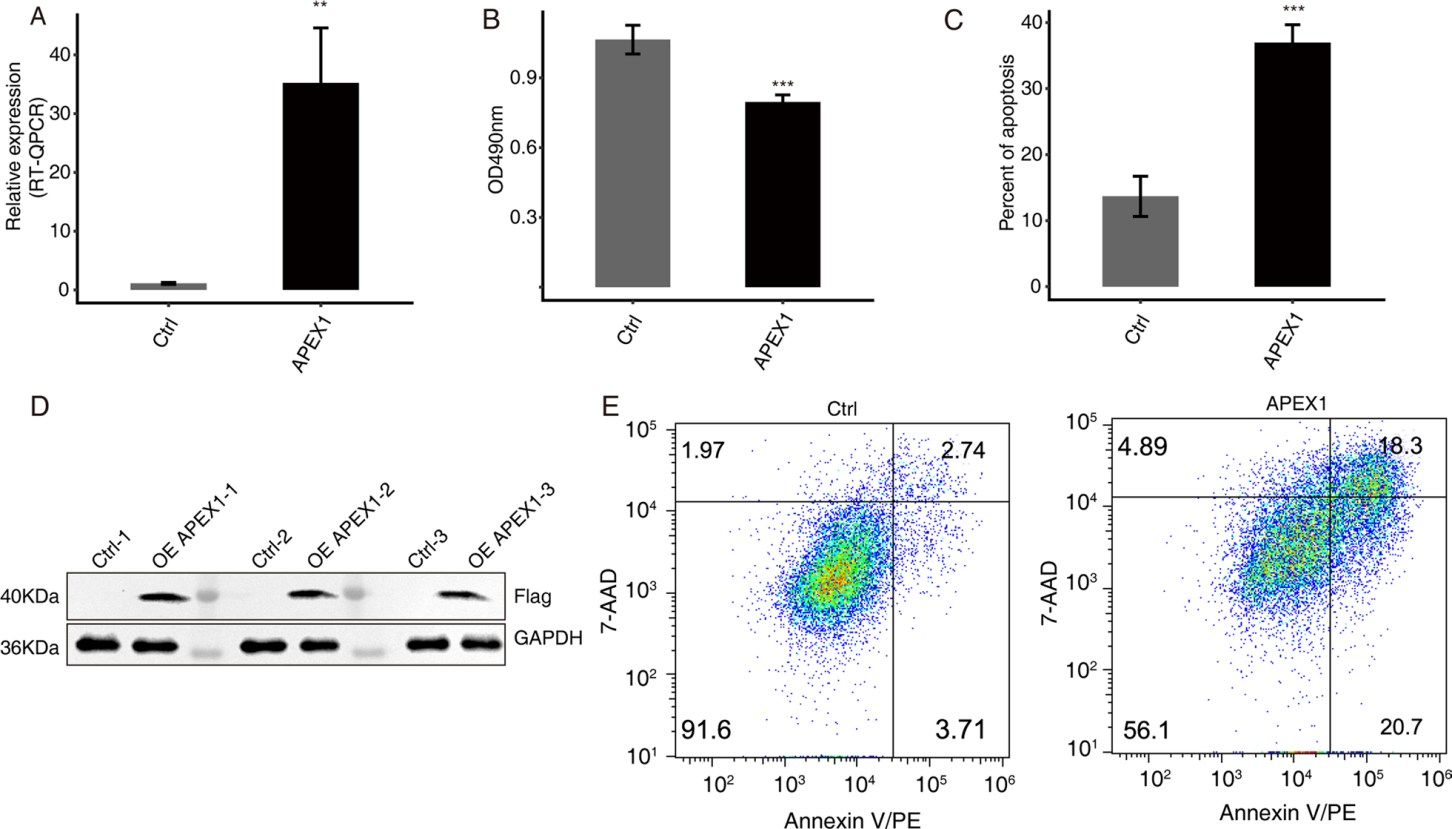
Effects of APEX1 on the proliferation and apoptosis of A549 cells. **a** Expression levels of APEX1 in A549 cells after transient transfection with APEX1 overexpression or control vectors, detected by RT-qPCR. **b** Cell proliferation analysis. **c** Apoptosis percentage of A549 cells transfected with APEX1 was quantified. **d** Western blot analysis showed high levels of APEX1 in cells transfected with the over expression vector vs. the control vector. **e** APEX1 overexpression induced A549 cell apoptosis. **p < 0.05, and ***p < 0.001

To analyze the effect of APEX1 on proliferation and apoptosis of cancer cell, A549 cells were transfected with control and APEX1 overexpression vectors (Fig. 2a Primer sequence information is shown in Materials and Methods). As shown in Fig. 2, over expression of APEX1 in A549 cells was confirmed by RT-qPCR and western blot analysis (Fig. 2a,d). Cell proliferation was significantly reduced by APEX1 overexpression when compared with the control (*p* < 0.05, Fig. 2b). Conversely, overexpression of APEX1 increased apoptosis of A549 cells (*p* < 0.05, Fig. 2c,e). These results demonstrated that overexpression of APEX1 reduced cell proliferation and promoted apoptosis in the NSCLC cell line, A549. The molecular mechanism for these effects requires further investigation at the molecular level.

### Transcriptome analysis and identification of APEX1-mediated AS of cancer-related genes in A549 cells

**Fig. 3:**
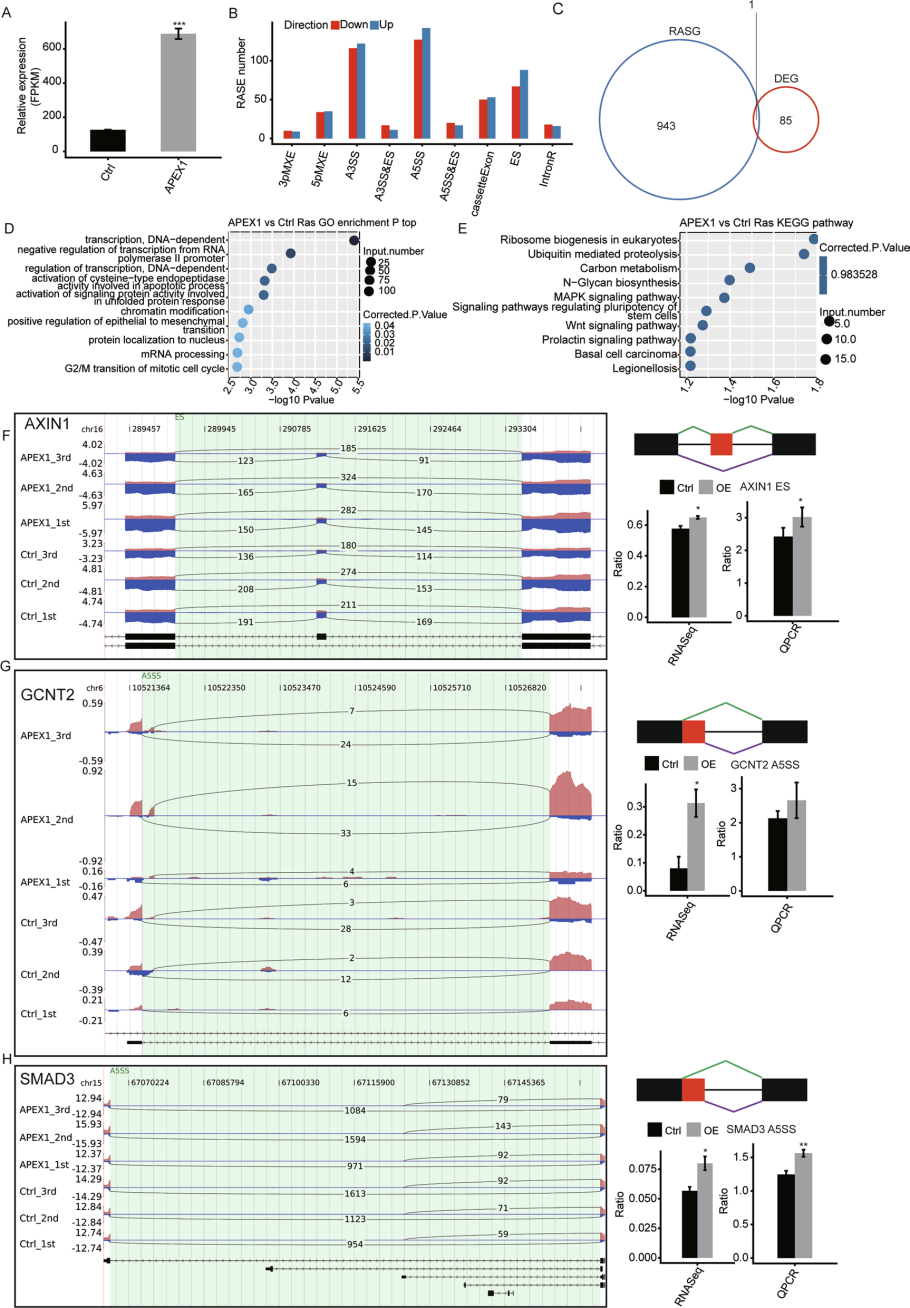
Identification and functional analysis of APEX1-regulated splicing events in A549 cells. **a** APEX1 expression quantification. **b** Classification of different AS types. **c** Overlap analysis between APEX1-regulated DEGs and RASGs. **d** The top 10 GO biological processes. **e** KEGG functional pathway analysis. **f**–**h** Splicing events genes validation. RNA-seq quantification and RT-qPCR validation of ASEs are shown at the bottom of the right panel. *p and **p < 0.05, ***p < 0.001

The successful overexpression of APEX1 was demonstrated by analyzing the FPKM (fragment per kb per million reads) values as detailed in the Materials and Methods (Fig. 3a). In order to gain insight into the role of APEX1 in AS regulation, we performed transcriptome sequencing of A549 cells with or without APEX1 overexpression. A total of 81 M ± 1.06 M uniquely mapped reads were obtained from APEX1-OE (overexpression) and control groups, in which approximately 49.75–52.92% splice events were junction reads (details can be found in Additional file 3: Table S3). Effective depletion of APEX1 was confirmed in parallel by RNA-seq analysis (Fig. 3a). We further explored APEX1-regulated AS events with the RNA-seq dataset using ABLas software. We detected 36,086 known ASEs and 55,088 novel ASEs, excluding intron retention (IR) (details can be found in Additional file 4: Table S4). We identified 611 high-confidence APEX1-RASEs. The complete RASEs can be found in Additional file 5: Table S5 and Fig. 3b. These data suggested that APEX1 regulated AS events. Furthermore, we analyzed genes whose transcription levels were regulated by APEX1-OE by running edgeR package, which resulted in 86 of such DEGs. As shown in Fig. 3c, only one RASG overlapped with a DEG.

To assess the potential biological function of APEX1-regulated AS, RASGs were subjected to GO and KEGG analysis. The top three GO biological terms were: activation of cysteine-type endopeptidase activity involved in apoptosis, regulation of transcription, and G2/M transition of mitotic cell cycle (Fig. 3d). The enriched KEGG pathways included: the MAPK signaling pathway and the Wnt signaling pathway (*p* ≥ 0.05, Fig. 3e and Additional file 6: Table S6). Furthermore, in order to validate APEX1-RASEs, q-PCR was performed. Out of 21 tested events, three alternative splicing events validated by q-PCR agreed with the RNA-seq results. The three validated splicing events were in the following genes, AXIN1 (axis inhibition protein 1), GCNT2 (N-acetyl glucosaminyl transferase 2), and SMAD3 (SMAD Family Member 3), in the Fig. 3f–h. In summary, these results demonstrate confidence in the APEX1 related RASEs and AS data.

### Validation of APEX1-regulated gene expression and alternative splicing in NSCLC clinical samples

**Fig. 4:**
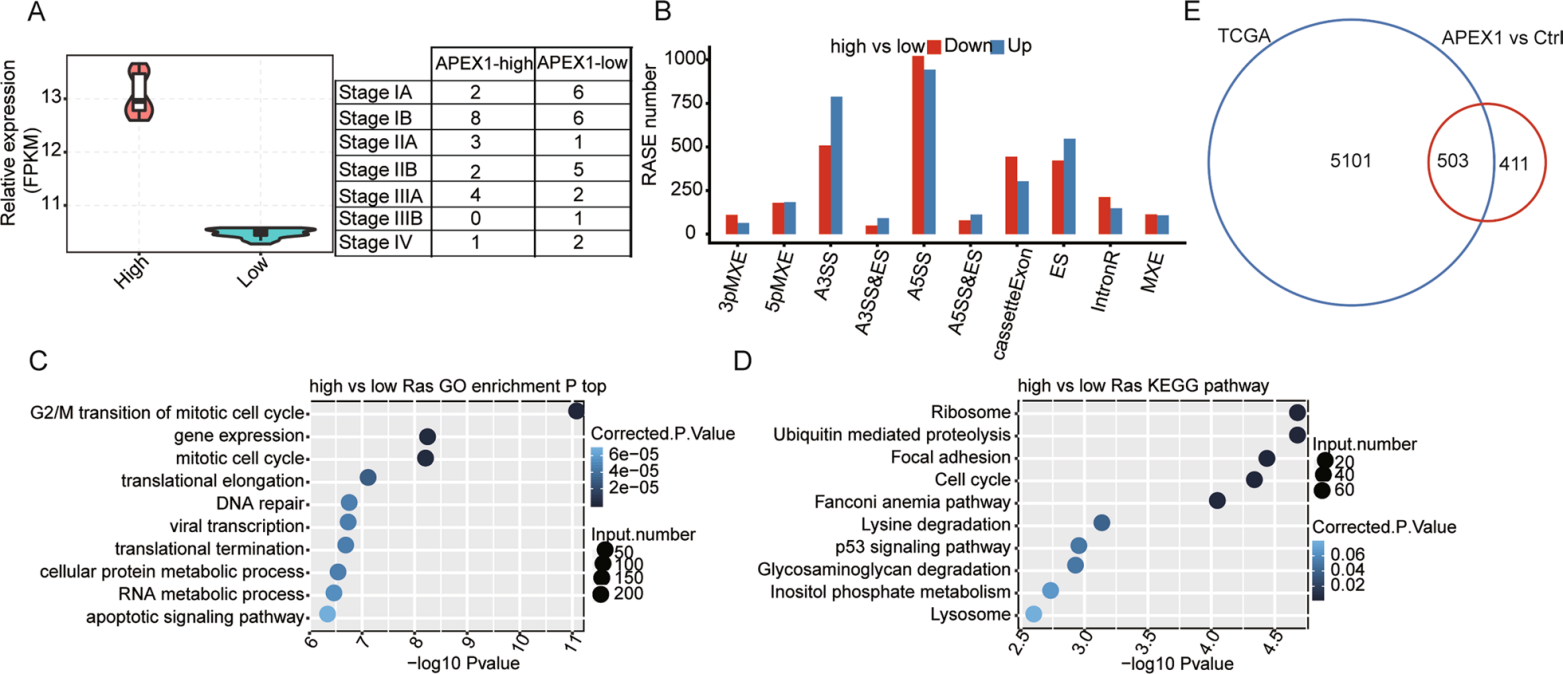
Identification and functional analysis of APEX1-RASEs and genes in NSCLC clinical samples. **a** Violin plot of APEX1 expression in two groups. **b** Bar plot of the distribution of ASEs. **c** The top ten GO biological processes. **d** KEGG functional pathway, **e** Venn diagram showing overlapped RASE genes *p < 0.05

In order to validate confidence in the DEGs and ASEs detected in this study, we performed additional experiments with NSCLC clinical samples from the TCGA Lung study. Forty cancer samples were selected from 20 high and 20 low APEX1 expression samples (Fig. 4a). Reads per sample were downloaded from TCGA database (details can be found in Additional file 7: Table S7). We identified 4626 high-confidence RASEs related to APEX1 expression levels in these 40 clinical samples (Fig. 4b). These data indicated that APEX1 extensively regulated ASE in NSCLC. Genes carrying APEX1-RASE were highly enriched in the following signal transduction pathways: apoptotic signaling pathway, cellular protein metabolic process, and G2/M transition of mitotic cell cycle (GO biological process terms, Fig. 4c). Meantime Enriched KEGG pathways (*p* ≥ 0.05) included; cell cycle, ubiquitin mediated proteolysis, and p53 signaling pathway (Fig. 4d). Taken together, these results indicate that APEX1-RASEs may play a role in NSCLC genesis. Because cancer tissues are complicated by multiple cell types and deregulated genes, these potential APEX1-RASEs could contribute to oncogenesis. Furthermore, to illustrate the consistency of AS between clinical samples and the A549 cell line, we carefully analyzed the expression levels of APEX1-RASs in NSCLC and A549 cells. We found overlap of the RASEs, with validation by RNA-seq and q-PCR (Fig. 4e).

## Discussion

APEX1, known as RBP, played a critical role in cancer [12, 19, 20]. The APEX1 overexpression is correlated with cancer progression in various human solid malignancies [21]. However, the involvement of APEX1 in cancer progression, especially its impact on genes within functional signaling pathways, has not been fully elucidated. The main finding of this study was that APEX1 was upregulated in human NSCLC tissues, and that APEX1 increased cellular apoptosis and decreased cellular proliferation by regulation of AS. Further, APEX1 affected key tumorigenesis genes of Wnt signaling pathway and MAPK signaling pathway by regulating AS. These genes included AXIN1, GCNT2, and SMAD3. Our results were confirmed in NSCLC tissues from the TAGA database and as well in A549 cells.

Previous study reported that certain patients with NSCLC who are eligible for newer targeted therapies or immunotherapies are now surviving longer, with 5-year survival rates ranging from 15 to 50%, depending on the biomarker. Therefore, a predictive biomarker has a corresponding specific targeted therapy that has been shown to improve outcomes in patients with the predictive biomarker (eg alectinib and brigatinib) [22]. Recent evidence has demonstrated APEX1 upregulation in various cancers such as NSCLC and colorectal cancer [23]. However, the clinical significance and concomitant role of APEX1 in cancer remains unclear, although APEX1 has been shown to be a positive regulator, contributing to the aggressive behavior of cancer behaviors. Wu et al. [24] knocked-down and overexpressed p53 in various lung cancer cells, their results showed that the cytoplasmic APEX1 expression elevated by p53 aberration may be used to predict poor survival and recurrence of NSCLC patients. In addition, as an emerging biomarker, serum APEX1 was found to be a predictive marker of platinum-based chemotherapy in NSCLS patients [25]. Furthermore, many studies have suggested increased APEX1 levels to be diagnostic and prognostic for cancer, and that APEX1 may be a therapeutic target for treatment of advanced cancer [26, 27]. Our research has analogous findings that APEX1 was cancer-promoting and that AS of cancer-related genes had a critical impact on lung cancer cell biology [28]. However, the specific mechanism by which APEX1 induces such effects requires further investigation.

Given the widespread AS perturbations in cancer, it is important to determine how AS events mediate cancer progress. Evidence has demonstrated that AS alterations in cancer can be caused by changes in expression, amplification, and deletion of RBPs [29, 30]. It has been established that, in some cases, a relationship exists between a splicing event and an increase in cancer cell proliferation and invasion [31, 32]. As such, AS may be a hallmark of cancer [33]. Our research found that AS and APEX1 were critical in the regulation of signaling pathways that control cell proliferation and tumorigenesis, including the MAPK and Wnt signaling pathways, which may be detrimental to NSCLC. The mechanism could be that APEX1 regulated AS of key tumorigenesis genes involved in cancer proliferation and apoptosis within the MAPK and Wnt signaling pathways, including genes encoding kinases and transcription factors. Our results further supported the concept that targeting differential AS related to APEX1 may be a means by which to reduce cell proliferation and induce apoptosis of tumors in patients with lung cancer. Further investigations are needed to elucidate the regulatory mechanisms by which AS impacts genes related to the MAPK and Wnt signal pathways. These investigations could provide for the development of new means by which to target malignant tumors.

In addition, by qPCR and RNA-seq we identified several cancer-related genes that regulated by AS of APEX1, which included AXIN1, GCNT2, SMAD3, CTBP2, PPARD, and FBXW11. Compared to the control group, these APEX1 related genes were upregulated and consistent with APEX1-RASEs identified. Mechanistically, we have demonstrated APEX1 inhibition to induce a rapid downregulation of AXIN1. AXIN1 is an important regulator of beta-catenin, which has been found in various human cancers [34]. A previous study found that reduced expression of AXIN1 was related to poor differentiation of lung cancer. Therefore, AXIN1 may provide a new target for therapeutic intervention in lung cancer. In this study, we found GCNT2 to be overexpressed in highly metastatic NSCLC samples. Previous functional studies showed that ectopic expression of GCNT2 enhanced cell migration and invasion in vitro, and lung metastasis of breast cancer cells in vivo [35]. So GCNT2 may be a novel gene contributing to metastasis with preferential expression in lung cancer. Furthermore, we found that APEX1 activated the TGFβ/SAMD3 signal pathway by promoting lung cancer cells proliferation**,** which may activate or repress their target gene promoters.

These results confirmed that APEX1 indirectly regulated development of NSCLC by affecting AS of the above-mentioned genes, thereby controlling proliferation and apoptosis-related signaling pathways. In point of fact, RBPs are overexpressed in a wide variety of cancers and the silencing of these genes induces apoptosis in cancer cells [36]. AS of cancer-related genes have a critical impact on lung cancer cell biology**.** Previous studies have found that alterations in genes encoding RBPs are pervasive in cancer, characterize different tumor types. Our results demonstrated, for the first time, that APEX1 played a very important role in the biology of NSCLC by regulating AS of key tumorigenesis genes related to proliferation and apoptosis signaling pathways. The APEX1 may play a role in promoting cancer by inducing the expression of many oncogenes, although identification of the precise mechanism requires further study.

However, several limitations of this study should be mentioned. Firstly, we collected 517 lung NSCLC samples detailed from the database and used the A549 cell line to verify our observations, but the specific mechanism by which APEX1 induces such effects requires further investigation. Secondly, this study focused attention on the functions of APEX1 in A549 cells due to the time and resource limitation. We will further verify and extend discovery in other cell lines in future studies. Finally, more functional researches will be performed in futures, which are important to confirm the biological relevance of APEX1-associated AS in lung cancer.

## Conclusions

In conclusion, our study successfully applied RNA-seq technology to demonstrate APEX1 regulation of AS. We demonstrated APEX1 expression was up-regulated in NSCLC samples, and that APEX1 over expression may reduce cell proliferation and induce apoptosis. In addition, we confirmed that APEX1 regulated the AS of key tumorigenesis genes involved in cancer proliferation and apoptosis pathways, mediating NSCLC progression. Further investigation to clarify molecular mechanisms is necessary. Additional studies in other cancer types may be investigated to test whether the modulation of AS by targeting APEX1 could be broadly applied as novel therapeutic strategy for cancer.

## Supplementary Information

**Additional file 1: Table S1**. The primers for detecting alternative splicing events (ASEs).

**Additional file 2: Table S2**. HiSeqV2 APEX1 expression box.

**Additional file 3: Table S3**. Mapping of clean reads on the reference genome.

**Additional file 4: Table S4**. All known AS events detected by type.

**Additional file 5: Table S5**. Classification of all RASE events between sample groups.

**Additional file 6: Table S6**. Ras KEGG pathway identify.

**Additional file 7: Table S7**. Mapping of clean reads on the reference genome.

**Additional file 8**: Figures Control and OE-APEX1 flag original, unprocessed version.

**Additional file 9**. The original western blotting image of OE-APEX1 flag.

## Data Availability

The datasets generated and/or analyzed during the current study are available in the GEO database, submission number GSE159360 (https://www.ncbi.nlm.nih.gov/geo/).

## Abbreviations

A3SS: Alternative 3’splice site
A5SS: Alternative 5’splice site
APEX1: Apurinic-apyrimidinic endonuclease-1
AS: Aberrant alternative splicing
ASEs: Alternative splicing events
AXIN1: Axis inhibition protein 1
CE: Cassette exon
CTBP2: C-Terminal Binding Protein 2
DEGs: Differentially expressed genes
DMEM: Dulbecco's modified eagle medium
DMSO: Dimethyl sulfoxide
ES: Exon skipping
FBS: Fetal bovine serum
FBXW11: F-Box and WD repeat domain containing 11
FPKM: Fragments per kilo base of exon model per million fragments mapped
GAPDH: Glyceraldehyde-3-phosphate dehydrogenase
GCNT2: N-acetyl glucosaminyl transferase 2
GO: Gene ontology
HRP: Horseradish peroxidase
IR: Intron retention
KEGG: Kyoto encyclopedia of genes and genomes
MEX: Mutually exclusive exons
5pMXE: Mutually exclusive 5'UTRs
3pMXE: Mutually exclusive 3'UTRs
NSCLC: Non-small-cell lung cancer
MTT: Methyl thiazolyl tetrazolium
OE: Overexpression
PPARD: Peroxisome proliferator-activated receptor
RASEs: Regulated alternative splicing events
RBPs: RNA-binding proteins
RIPA: Radio immunoprecipitation assay
RT-qPCR: Quantitative reverse transcription polymerase chain reaction
SDS-PAGE: Sodium dodecyl sulfate polyacrylamide gel electrophoresis
SMAD3: SMAD family member 3
TCGA: The cancer genome atlas
