# Supplementary figures and images for: APEX1 regulates alternative splicing of key tumorigenesis genes in non-small-cell lung cancer
Source: BMC Med Genomics. 2022 Jul 2;15:147. doi: 10.1186/s12920-022-01290-0 (PMC9250739; doi:10.1186/s12920-022-01290-0)

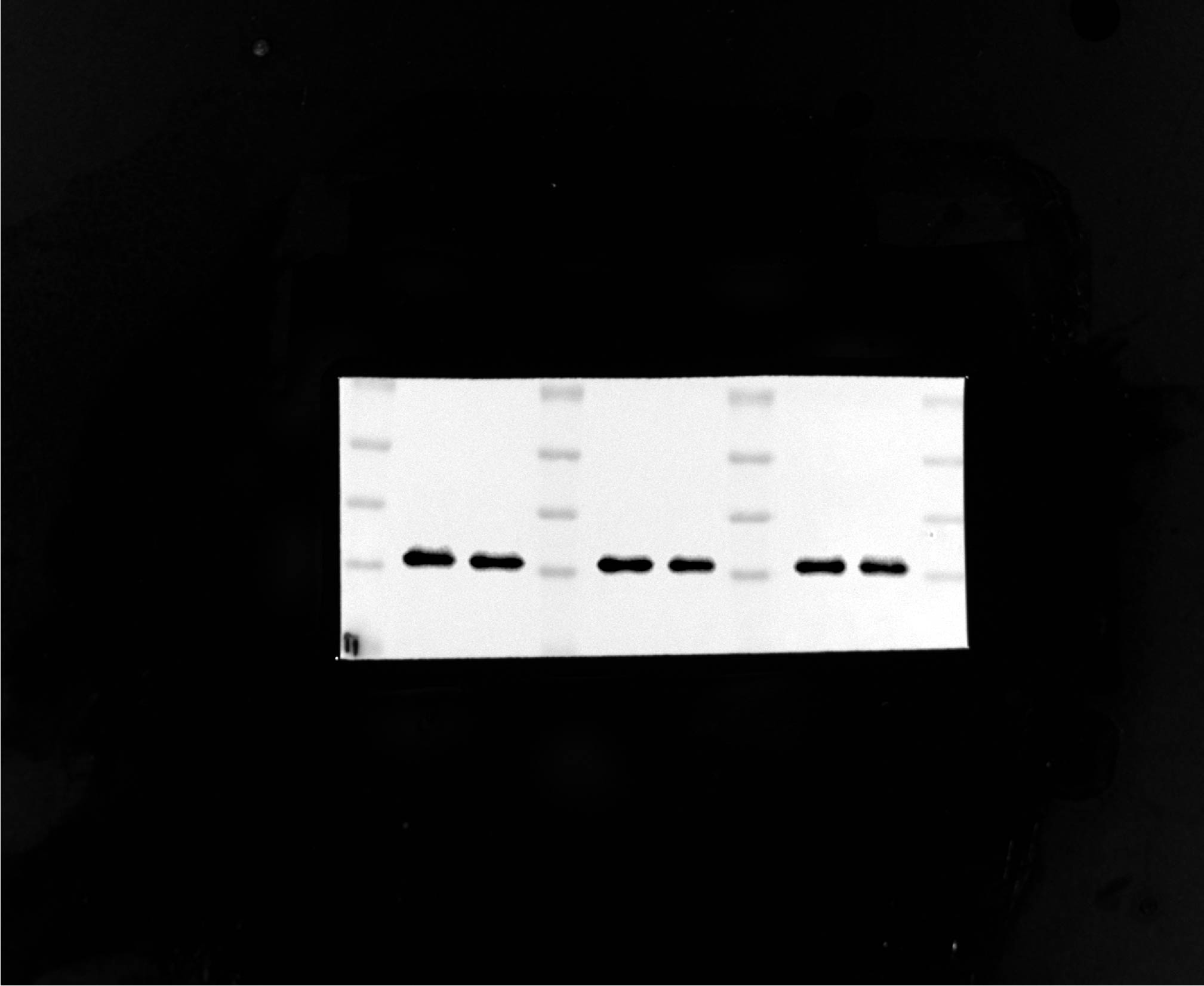

Supplement: Supplementary file 8 — Additional file 8: Figures Control and OE-APEX1 flag original, unprocessed version. [file 12920_2022_1290_MOESM8_ESM.jpg]

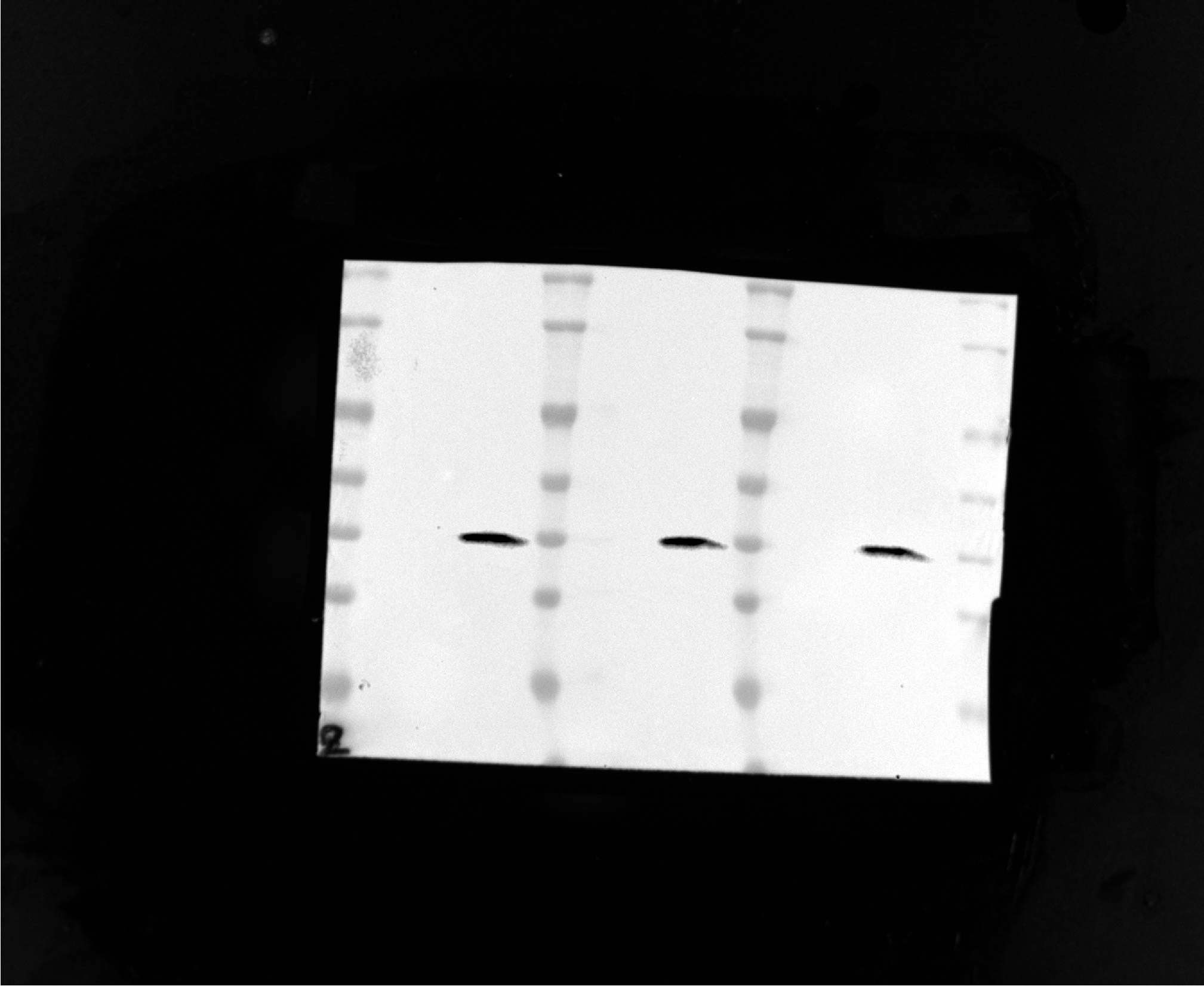

Supplement: Supplementary file 9 — Additional file 9. The original western blotting image of OE-APEX1 flag. [file 12920_2022_1290_MOESM9_ESM.jpg]
